# Querying archetype-based EHRs by search ontology-based XPath engineering

**DOI:** 10.1186/s13326-018-0180-2

**Published:** 2018-05-11

**Authors:** Stefan Kropf, Alexandr Uciteli, Katrin Schierle, Peter Krücken, Kerstin Denecke, Heinrich Herre

**Affiliations:** 1 Institute for Medical Informatics, Statistics and Epidemiology (IMISE), Leipzig University, Härtelstraße 16-18, Leipzig, 04107 Germany; 2 Institute of Pathology, Leipzig University Hospital, Liebigstraße 26, Leipzig, 04103 Germany; 3 Institute for Medical Informatics, Bern University of Applied Science, Quellgasse 21, Biel, 2501 Switzerland

**Keywords:** Electronic health records, Medical informatics applications, Search ontology, Information retrieval, EHR query, Pathology electronic health records, Query engineering

## Abstract

**Background:**

Legacy data and new structured data can be stored in a standardized format as XML-based EHRs on XML databases. Querying documents on these databases is crucial for answering research questions. Instead of using free text searches, that lead to false positive results, the precision can be increased by constraining the search to certain parts of documents.

**Methods:**

A search ontology-based specification of queries on XML documents defines search concepts and relates them to parts in the XML document structure. Such query specification method is practically introduced and evaluated by applying concrete research questions formulated in natural language on a data collection for information retrieval purposes. The search is performed by *search ontology-based XPath engineering* that reuses ontologies and XML-related W3C standards.

**Results:**

The key result is that the specification of research questions can be supported by the usage of *search ontology-based XPath engineering*. A deeper recognition of entities and a semantic understanding of the content is necessary for a further improvement of precision and recall. Key limitation is that the application of the introduced process requires skills in ontology and software development. In future, the time consuming ontology development could be overcome by implementing a new clinical role: the *clinical ontologist*.

**Conclusion:**

The introduced Search Ontology XML extension connects Search Terms to certain parts in XML documents and enables an ontology-based definition of queries. Search ontology-based XPath engineering can support research question answering by the specification of complex XPath expressions without deep syntax knowledge about XPaths.

## Background

### Precise questions on semi-structured medical records

Since clinicians prefer narratives and dictated speech over rigid entry forms [1], Electronic Health Records (EHRs) are often stored as free text. This information type is referred to by the term semi-structured, preassumed the documents are structured by headers and keywords manually assigned by the physicians. This structure is usually not technically implemented. Queries on such data can not be very precise because there is no semantic information explicitly available as markup in the free text.

In order to specify precise queries on semi-structured health records, a transformation of semi-structured health records into *Structured EHRs* is required as well as methods for *Querying on Structured EHRs*.

### Structured EHRs

“A well written patient history may be a narrative or structured document.[...] There is a drive to structure and/or code all clinically relevant information in EHRs to benefit from computability of information” [2]. Not only machines, also physicians are benefiting by structured documents, because “it seems that having an expectation of what to find under a certain heading makes for a faster interpretation of the text” [3]. Anyway, there are narrative as well as structured EHRs; and when the physicians structure their information using certain keywords and headers in the narratives, it is possible to transfer free text based medical records into standardized and section-structured XML EHRs [4]. Querying EHRs by keywords in certain sections requires that the sections are recognized by Section Boundary Detection (SBD) and stored in an appropriate format. In previous work [4], we showed, that such a transfer is possible: A set of pathology reports has been automatically transformed into archetype-based Pathology Electronic Health Records (PEHRs). The standard openEHR was exploited for this transformation.

### Querying structured EHRs

After the transformation process, queries can be applied to specific sections instead of the entire document. This can reduce false positive results. There is a need for an ontology-based way for the generation of XPath expressions. This method, referred to as *search ontology-based XPath engineering*, will be introduced in this work. More specifically, the suggested approach [5] will be proven in a real world scenario by real Research Questions (RQs) on a real data set. One hypothesis of this paper is: when the PEHRs are structured into sections by SBD and stored in an XML database, the sections can be used for Research Question Answering (RQA).

### Related work

Related work can be distinguished in *EHR Query Languages on Data Marts*, and *Ontology-based Queries*.

**EHR Query Languages on Data Marts** Particularly in health care, secondary use and mining on EHRs is still challenging [6]. There are already well defined query languages for archetype based EHRs [7, 8]. These query languages define an abstract language, which borrows keywords from Structured Query Language (SQL) [9], and combines them with archetype path expressions, which are similar to XPaths [10]. Another prominent SQL based approach is the usage of the i2b2 [11] data mart for querying EHRs. Precondition for that is an Extract Transform Load (ETL) transformation process into the i2b2 Star Schema [12].

**Ontology-based queries** When the data is stored on a structured relational database, semantic searches can be applied for answering different kinds of RQs [13]. The PONTE platform [14] enables querying on a global EHR ontology using SPARQL statements [15]. A similar approach uses ontology-based mediation and Object Query Language (OQL) for query formulation [16]. The XOntoRank system [17] enables semantic search by inferring semantic relationships between the query keywords and the terms in the documents (based on domain ontologies like Systematized Nomenclature of Medicine (SNOMED)). A promising approach is the SPARQL2XQuery framework [18], which enables both, transformation between XML and ontologies, and the query translation of SPARQL to XQuery [19].

**Reducing ETL processes** All in all, for answering RQs by structured query languages like SQL or SPARQL time consuming ETL processes are necessary. In essence, EHRs have to be transformed into data marts like i2b2 or an ontology for enabling SPARQL. Moreover, the transformation into data marts or ontologies requires structured data, but again, many EHRs consist of free text. We can skip these time consuming processes when queries are directly applied to PEHRs (using SBD and XPaths).

**Demarcation to Question Answering (QA) systems** Researching QA systems was an early explored research field in computer science [20]. Nowadays the topic of semantic QA systems is a comprehensive and active research field with many different approaches [21]. Nevertheless the approach of this paper can support experts during RQA by ontology-based query formulation and query generation, we distance this approach from general QA systems, because “QA systems directly return answers, rather than documents containing answers, in response to a natural language question” [22].

**Other limitations**

The category *Ontology-based Queries* is promising a higher precision than queries by keywords in certain sections, because SPARQL queries on OWL based patient data would be more powerful than XPath expressions on XML; but a comprehensive and long term persistence storage of pathology data within semantic web technologies is only partially solved. A deep semantic understanding of free text based EHRs is an open research topic, but in the near future especially the time consuming manual review process could be supported by methods of Named Entity Recognition (NER) and ontology extraction (→ “Discussion” section).

Generally speaking, the approach of this paper is inherent independent from the underlying XML structure and belongs to the category of *Ontology-based Queries*. We suggest the usage of an ontology, which is strongly bound to the used XML structure for the generation of XPath expressions. This strong binding on a structure is only meaningful when standardized XML-based EHRs are used.

### Approach and paper overview

We consider in this work RQs from the pathology domain as a concrete example (→ “M1. Questions by a domain expert” section) which have to be answered by a set of PEHRs. These PEHRs are stored after applying SBD to the (free) text on an XML database (→ “M2. structured PEHRs” section). After that, XPath expressions can address certain parts of the XML documents (→ “Querying PEHRs using XPaths” section). The development of such XPaths is time consuming for domain experts, but also for computer scientists. We suggest to use ontologies to support experts for answering RQs by *search ontology-based XPath engineering* (→ “I. SO-based XPath engineering” section) using the Search Ontology XML extension (SOX). For answering clinical RQs or for searching similar cases, XPaths can be generated automatically out of this ontology (→ “II. Automatic XPath generation” section), which in turn can be applied to document corpora on XML database systems.

**Fig. 1:**
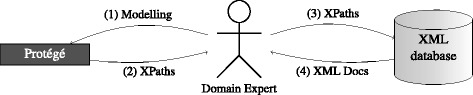
Use case overview: search ontology-based XPath generation

Figure 1 gives an overview of the idea of this paper. In the middle of the search process is a domain expert. On the left hand side of Fig. 1 it is illustrated, that the agent uses Protégé, the ontology editor of the Stanford University [23] for modeling the query using the SO (→ “Ontologies” section) and SOX (→ “Ontologies” section). On the right hand side of Fig. 1 the agent interacts with the XML database; by using XPaths (→ “Querying PEHRs using XPaths” section) the agent can retrieve relevant XML documents. In summary, focus of this work is the evaluation of the SOX-approach by trying to support RQA. The main contribution is a tool which is able to generate XPaths expressions out of the SOX (→ “Search Ontology XML Extenstion XPath Generator (SOXPathGen)” section). The tool is tested on sample PEHRs files (→ “Simple Test Files (Pathology Electronic Health Records” section) by applying five real-world RQs (→ Table 1).

**Table 1:** NL description of the queries (→ “Search Ontology-based Pathology Questions (OWL)” section)

Q	Question
*Q0* ^a^	*PEHRs which contains T2 as primary tumor classification*
	*and defined phrases of excised skin material*
Q1	Prostatic carcinomas are found starting from how many
	grams of flake tissue?
Q2	Prostatic carcinomas are found starting from how many
	capsules? What influence has the processing method
	(with/without remainder)?
Q3	How large are the leiomyomas of the uterus in the entry
	material?
Q4	How many lymph node metastasis occur at colon cancer
	in stage pT2?
Q5	In how many esophageal biopsies is a barret mucosa found?
	Exclude a certain negation expression^b^ (cave).

^a^Q0 is only for proofing the concept [5]

^b^’ohne Nachweis einer Barrett-Schleimhaut’ (en: without evidence of barrett mucosa)

## Material

### M1. Questions by a domain expert

Table 1 lists the questions in Natural Language (NL), that are asked by a pathologist, which we will try to solve by applying SOX. In this paper, the Question 1 (Q1) will be picked as continuous example, which will be referenced in the following sections. In Q1 the pathologist is interested in the average flake weight, that occurs when prostate cancer is diagnosed. More precisely:



Q1 is in principle a simple question, but it shows that processing NL questions is difficult to understand for humans as well as for machines. Because of that we are convinced: there is a demand of an ontological-based query formulation.

### M2. structured PEHRs

**Fig. 2:**
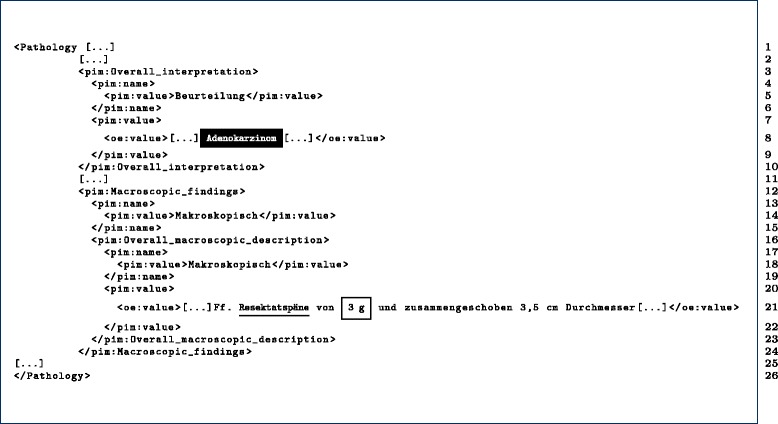
Simplified XML-based pathology EHR snippet, containing a specimen, an overall interpretation and a macroscopic findings part

In this article, we will concentrate on the special domain of pathology, where a lot of semi-structured information occurs in terms of pathology reports. In fact, pathology reports are based on certain section patterns and section-introducing keywords, like material, macroscopy or microscopy. We verified manually, that keywords like Material, Makroskopie or Mikroskopie were constantly used for section tagging of pathology reports of the Institute of Pathology of Leipzig. Therefore, the reports can be section-structured very precisely into an archetype-based Pathology Patient Information Model (PPIM) by the application of methods like SBD and openEHR [4]. As a result of this previous work, 68,583 openEHR-based PEHRs are stored on an XML database, ready for answering RQs. For a better understanding, we publish herewith some test files (→ “Simple Test Files (Pathology Electronic Health Records)” section). The corresponding XML of one sample PEHR is listed in Fig. 2.

## Methods

### Querying PEHRs using XPaths

**Fig. 3:**
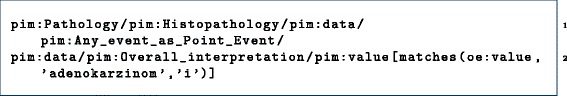
One simple XPath example

When EHRs are stored in XML, another query language is more suitable than classical free text retrieval methods such as Lucene [24]. XPath expressions are following the structure of the EHRs and are a W3C standardized method for addressing parts in XML documents [10]. An example XPath expression regarding Q1 is shown in Fig. 3. XPath functions are used for matching the German word stems. E.g. when ’florid(∖w)*’ is used as matching pattern, we will also find any variation like ’floride’ or ’florides’. Of course, irregular words needs to be treated by multiple disjunct specifications. For the combination of words, the expression ([ ∖w]* ∖s){0,2} can be useful, which implies that a maximum of two words is allowed to match the pattern, which is similar to Lucene Proximity Searches [24].

### Ontologies

**Fig. 4:**
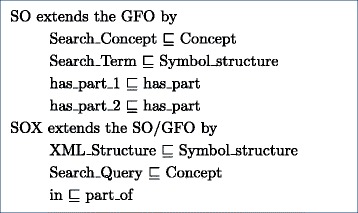
SO → SOX

**Top level ontology General Formal Ontology (GFO)** The GFO introduces a top level ontology [25], useful for conceptual modeling. The GFO classes Concept and Symbolic_structure and the property has_part have been reused during the introduction of the SO and SOX classes and properties (summarized in Fig. 4).

**Fig. 5:**
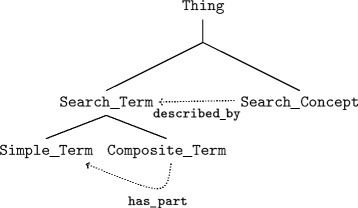
Overview search ontology

**Search ontology** The development, management and reuse of search concepts is a complex task, that can be supported by the SO [26]. The SO has been developed to support full text search on documents; it can be used for Information Retrieval (IR) in any domain by extending it by the corresponding domain ontology. The representation of the knowledge in the SO is similar to knowledge-based IR, where Hierarchical Concept Graphs (HCGs) constitute hierarchical thesauri as an useful knowledge representation [27]. In the SO we distinguish Search_Concepts from Search_Terms, disaggregating the latter into Simple_Terms and Composite_Terms. Composite_Terms are made up of Simple_Terms, related by the Object Property has_part, and Composite_Terms are constrained by the additional data property max_distance, which defines the word distance between Simple_Terms, where max_distance=0 represents, that one word immediately follows another word. Writing variations, synonyms, abbreviations as well as term phrases can be handled by the assignment of multiple labels to the concrete individuals of a Simple_Term. The SO is illustrated and described in detail in Fig. 5.

**Fig. 6:**
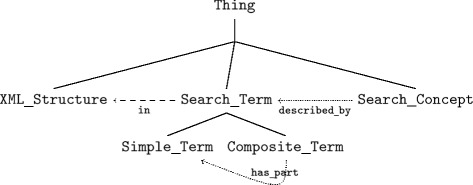
Search ontology XML extension

**Search ontology XML extension** We extended already the SO in a way that allows querying structured data stored as XML documents [5]. By extending the SO, XPaths are automatically producible out of the ontology, which can be executed on XML documents by integrating them into XSLT or XQueries. The extension of the SO is summarized in Figs. 4 and 6. On the top level of the ontology the class XML_Structure was added, which subclass structure represents the XML structure. Figure 6 shows that Search_Concepts are described_bySearch_Terms. Search_Terms belong to certain parts in the XML_Structure, linked by the added in relation. Namespaces and tag names of the XML document are defined within the class IRI. For a combination of multiple Search_Concepts, we enhance the SO by a new class, the Search_Query (→ “I. SO-based XPath engineering” section). Further, an additional annotation property xpath is adhered during the XPath generation process (→ “II. Automatic XPath generation” section).

### Engineering and generation process overview

**Fig. 7:**
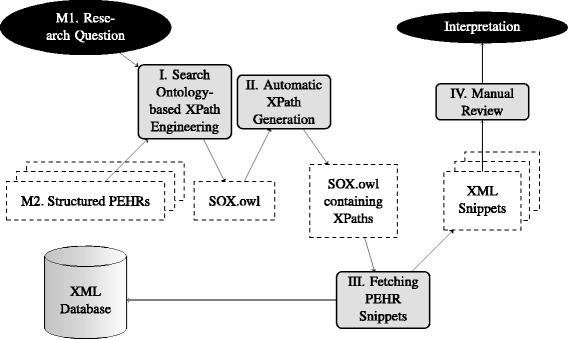
Overall process overview

Figure 7 is important for understanding the overall process, in which the ontology methods are used. Prerequisite for the query engineering is a concrete RQ (M1) and structured PEHRs (M2), which are stored on an XML database. The process illustrated in Fig. 7 is described in the following subsections (I.-IV.).

#### I. SO-based XPath engineering

The modelling of the queries has to be done manually and consists of the following sub-steps:

- Defining the XML_Structure
- Understanding and Formalization of the Questions
- Preparing the Search_Terms
- Describing the Search_Concept and linking them to the XML_Structure
- Combining Search_Concepts to Search_Queries

The process order is not strict. In practice, it is also useful to describe the Search_Concept (I.4) before the definition of the Search_Terms (I.3). Practical query engineering is a cyclic process (→ “Uncertainty of NLs” section), which will be explained in the following by a practical example.

**Fig. 8:**
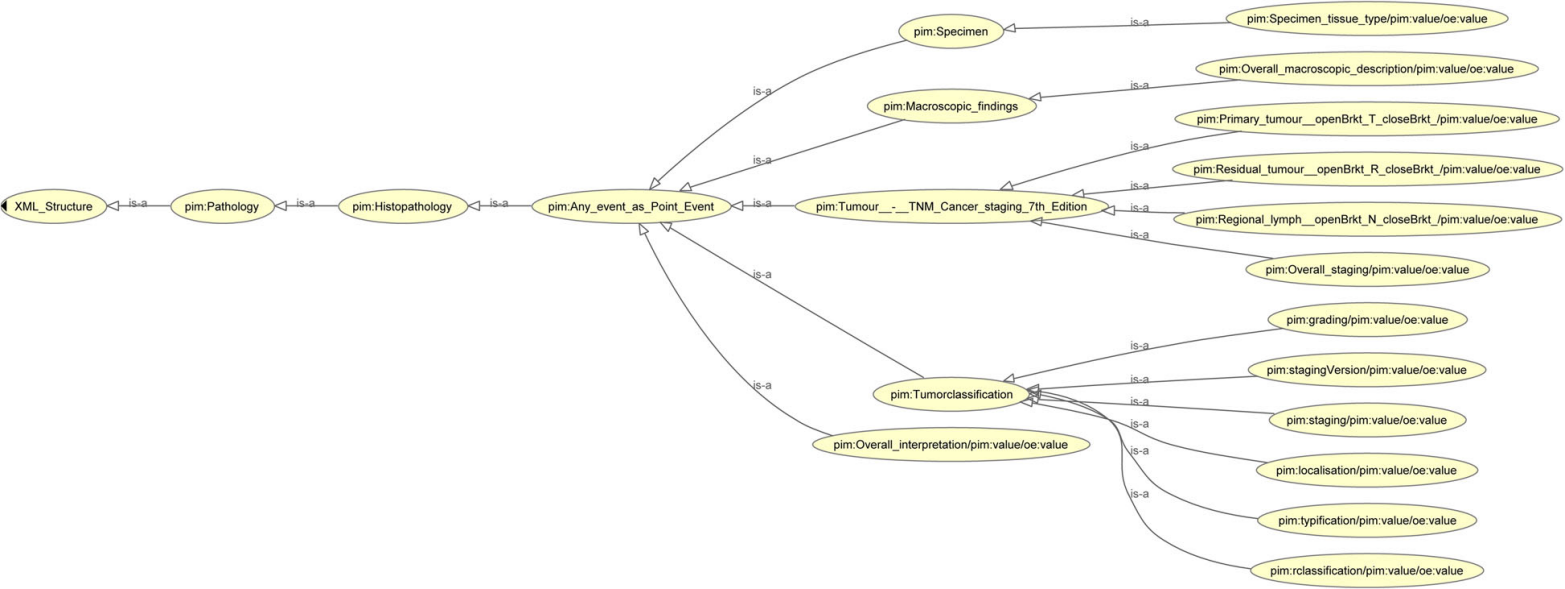
XML_Structure tree

**I.1 Defining the XML_Structure** The definition of the XML_Structure in a HCG is conditional, because Search_Terms have to be bound to the XML_Structure in a later sub-step. Namespace declarations are directly used in the IRI. Figure 8 illustrates the XML_Structure, which is based on the PEHRs and required for answering the questions of Table 1.

**I.2 Understanding and formalization of the questions**

In this preparation step, all questions of Table 1 can be formalized like suggested in Table 2. Another approach would be the usage of NL, as long as it is clear and complete.

**Table 2:** DL-based-description of the queries

Q	Question
*Q0* ^a^	(**HE_Shapes***inMacroscopy AND*
	**T2_Term***in Overall_staging*) [5]
Q1	**G_Unit***in* Macroscopy *AND* ¬**Blister**
	*in* **Interpretation** *AND*
	(**Adenocarcinoma***in* Interpretation *OR*
	(**ICD-O-C-61***in* Localisation *AND*
	**ICD-O-M-8140/3***in* Typification))
Q2 (without residual)	**K_No_Rest***in* Macroscopy *AND*
	¬**Blister***in***Interpretation***AND* (**Adenocarcinoma**
	*in* Interpretation *OR* (**ICD-O-C-61***in* Localisation *AND*
	**ICD-O-M-8140/3***in* Typification))
	*AND* (**ProstateFlake***in***Macroskopy**)
	*OR***ProstateFlake***in***Interpretation**)
Q2 (with residual)	**K_Rest***in* Macroscopy *AND* ¬**Blister**
	*in* Interpretation *AND*
	(**Adenocarcinoma***in* Interpretation
	*OR* (**ICD-O-C-61***in* Localisation *AND*
	**ICD-O-M-8140/3***in* Typification)) *AND*
	(**ProstateFlake***in***Macroskopy***OR*
	**ProstateFlake***in***Interpretation**)
Q3	**CM_Unit***in* Interpretation *AND***Leiomyom***in*
	Interpretation *AND***Uterus***in* Material
Q4	(**C18***in* Localisation or Colon *in* Material) *AND***T2***in*
	Overall_staging *AND***TNM_Sub_pN***in* staging
Q5 (numerator)	**BarrettsMucosa***in* Overall_interpratation *AND*
	**NO_Exclusion_Cave***in* Interpretation
Q5 (denominator)	**EsohagusBiopsy***in* Material

^a^Q0 is only for proofing the concept [5]

The *in* relation was introduced in SOX. **X***in* Y means that at least one instance of the *Search_Term* class **X** (bold) should occur in the section representing class Y

**Fig. 9:**
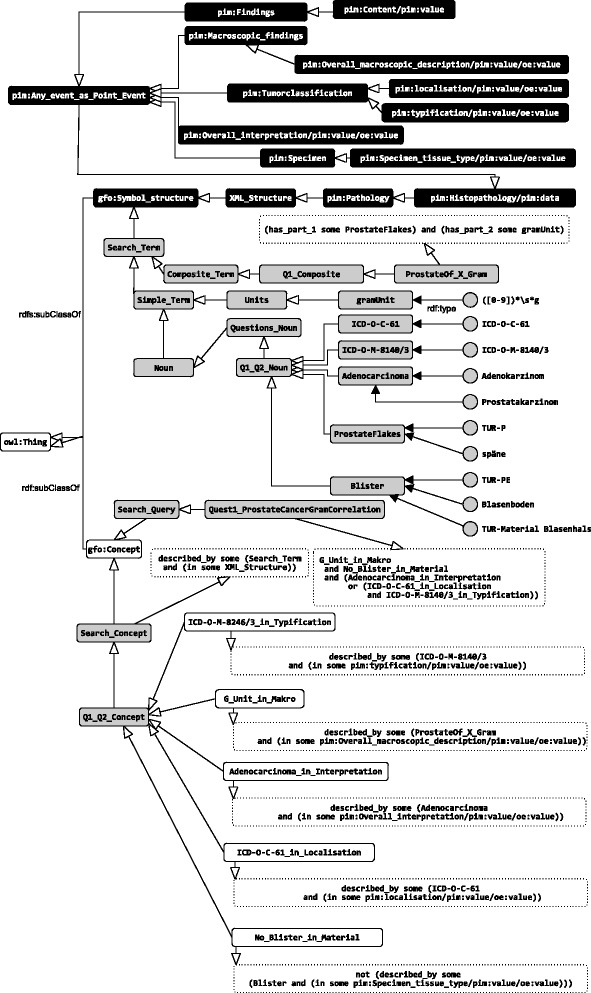
Class Quest1_ProstateCancerGramCorrelation

**I.3 Preparing the Search_Terms** Based on the latter sub-step (Table 2) the Search_Term classes, more precisely Simple_Terms and Composite_Terms, were defined. Firstly Simple_Terms classes and instances were defined; multiple labels can be created, which can contain regular expressions. Figure 9 illustrates the defined Search_Term classes and labels regarding Q1. After defining the Simple_Terms, Composite_Terms can be constructed by linking them to the Simple_Terms by the has_part relation.

**I.4 Describing the Search_Concept**

Search_Concepts are primitive classes, which are described by the following someValueFrom restriction:



For instance (Q1), to refine a Search_Concept to a class which represents, that certain adenocarcinoma Search_Terms are expected in an Overall_interpratation section, the following class description is used.



**I.5 Combining Search_Concepts to Search_Queries** It became clear during the engineering process of this practical use case, that an additional concept is needed for connecting multiple Search_Concepts together by Boolean expressions. The following class description represents the combination of multiple Search_Concepts regarding Q1.



There is an improved readability when we compare *(1) Query for answering Q1 in NL* with the latter *(2) Q1 class description*.

#### II. Automatic XPath generation

The latter ontological query engineering yields an OWL file, that holds all necessary data for the automatic generation of the XPath expressions. During that generation, each Search_Query concept gets an XPath annotation. These annotations are generated by a program fetch, that interprets the class descriptions and labels by the usage of the Jena API [28]. The algorithm dissolves each Search_Concept contained in the Boolean expression of each Search_Query. When the Search_Concept is described_by a Simple_Term, a disjunction is generated, that contains for every instance label of the Simple_Term an XPath expression; the generation is based on the labels of the Simple_Term instances and is based on the path of the referenced XML_Structure node. Otherwise, when the Search_Concept is described_by a Composite_Term, a disjunction of a constructed cross product of the referenced Simple_Terms is generated.

#### III. Fetching EHR snippets

The generated XPath expressions are integrated in XQueries, which are applied on an XML database for retrieving relevant XML snippets. After that, the relevant PEHR snippets are stored on the local file system, ready for the manual review.

#### IV. Manual review

During the manual review process, the retrieved PEHRs snippets have to be evaluated and interpreted. Ideally after that step, the initial RQ can be answered. In practice circles occur, which means that the question has often to be refined during the manual review.

## Results

The main contribution of this work, the introduced method *SO-based XPath engineering*, has been evaluated by the application of the described process by an ontologist, where five RQs have been processed. Each process yields interim results, that will be presented in the following. Based on these interim results, which are OWLs and PEHR snippets, a short interpretation of the RQA indicates the practical usefulness of the presented approach.

### I. SO-based XPath engineering and automatic XPath generation

**Fig. 10:**
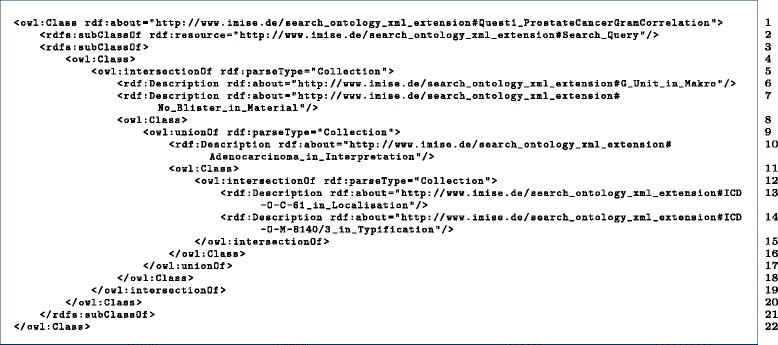
OWL Class Quest1_ProstateCancerGramCorrelation

The OWL class descriptions (which relate to Q1) are verbosely listed in Fig. 10. For a better understanding, we published the resulting OWL files containing

- the generated XPath expressions for the five RQs (→ “Search Ontology-based Pathology Questions (OWL)” section),
- as well as the binary of the XPath generation tool (→ “Search Ontology XML Extenstion XPath Generator (SOXPathGen)” section).

### II. Fetched PEHR snippets and manual review

The XPaths have been applied within XQueries for fetching the relevant PEHR snippets. The second column of the Table 3 shows the amount of retrieved XML snippets for each of the five questions. These PEHR snippets are used for RQA during the manual review, where each PEHR snippet has to be evaluated to prevent false positives in the query result. After removing the false positives, the PEHR snippets are ready for the interpretation.

**Table 3:** Overview on the evaluation results

Question	|*PEHR*|	|*PEHR*|	|*PEHR*|	|*PEHR*|
		(partly) enumerated	ECRI false	PQCRI false
			positives	positives
*Q0* ^a^	*12*	*9*	*n/a* ^b^	*n/a* ^c^
Q1	36	5	1	0
Q2 (without residual)	18	6	0	0
Q2 (with residual)	9	2	0	0
Q3	153	67	1	60
Q4	4	4	n/a^b^	*n/a* ^c^
Q5 (denominator)	902	632	*n/a* ^d^	*n/a* ^c^
*Q5* (numerator)	*756* ^e^	*skip* ^f^	*n/a* ^d^	*n/a* ^c^
Sum^g^	1134	725	2	60

^a^Q0 is only for proofing the concept [5]

^b^not structured by an enumeration list, TNM classification codes are used

^c^PQCRI can not occur because no units are used in this query

^d^ECRI can not occur because in this type of PEHR the specimen tissue section was not structured by an enumeration list

^e^not part of the column sum because Q5 (denominator) contains the Q5 (numerator) records

^f^evaluation can be skipped because Q5 (denominator) contains already the Q5 (numerator) records

^g^without Q5 (numerator)

In the second column is the amount of the retrieved PEHRs, in the third column is the amount of numbered content, in the fourth column is the amount of false positives which occur because of the ECRI, and in the fifth column is the amount of false positives which occur because of the PQCRI

### III. Interpretation

Table 3 summarizes the amount of retrieved PEHRs and indicates the counts of cases of enumerated content. In the result set, about ≈64*%* of the PEHRs contained enumeration lists. Moreover, all RQs of Table 1 could been answered in Table 4. In particular, the amount of results retrieved for Q1, Q3, and Q5 are useful for answering the corresponding RQs:

**Table 4:** Answers of the NL Questions based on the dataset of 68,583 PEHRs, interpretated by the ontologist

Q	Answer
Q1	The least weight was 3 *g*, the maximum weight
	was 38 *g*, were prostate carcinomas have been
	found. The average weight was
	≈18.26 *g*, *σ*≈10.18 *g*.
Q2 (without residual)	At least 2, at most 26 *capsules* were took without
	rest. In average 9.28 *capsules* were took,
	*σ*≈4.78 *capsules*.
Q2 (with residual)	At least 6, at most 10 *capsules* were took with rest.
	In average ≈9.55 *capsules* were took,
	*σ*≈0.15 *capsules*.
Q3	≈2.76 *cm* is the maximum diameter of leiomyomas
	in average, *σ*≈1.42 *cm*.
Q4	In four found cases^a^ 0.5 *metastasis* occur at colon
	cancer in stage pT2 in average.
Q5	In 83.81*%* of the esophageal biopsies a barret
	mucosa has been found.

^a^(1/1), (1/1), (0/41), (0/19)

- The average weight of flakes ≈18.26 *g* seems to be reasonable.
- Especially the relatively high amount of 93 cases indicates, that the average maximum diameter of leiomyomas of ≈2.76 *cm* could be a plausible answer.
- The high amount of cases indicates, that in about 8 of 10 cases a barret mucosa has been found during an esophageal biopsy. This value is a characteristic quality factor, usable for a comparison of clinicians as well as institutes.

All questions could be better evaluated by a bigger amount of PEHRs in the database.

## Discussion

We introduced an extension of the Search Ontology to support querying XML documents. The SOX approach can simplify the generation of a big pool of XPath expressions. During the practical evaluation of the approach, difficulties regarding NL arose, which will be discussed in the following.

### Uncertainty of NLs

**Uncertainty of NL questions** Q1 can be interpreted in different ways: (1) The pathologist wants to know the minimum known flake weight, were prostate carcinoma could be diagnosed. (2) The pathologist wants to know an avarage value. (3) The pathologist wants to know a value range. We solved this uncertainty by offering answers of all of these variations in Table 4.

**Uncertainty in the material** During the manual review process, we recognized a frequent occurrence of certain types of false positives in the result set: *(1) Enumeration Coreference Resolution Issue (ECRI)* and *(2) Physical Quantity Coreference Resolution Issue (PQCRI)*.

**(1) ECRI** In essence, an enumerated PEHR consists usually of different *material* items: *mat*_1_, …, *mat*_*i*_, *mat*_*n*_; and then, the *macroscopy* section could also have an enumeration list *mac*_1_, …, *mac*_*j*_, *mac*_*n*_. Imagine we found a PEHR, where *mat*_*x*_ contains one related search term (e.g. ’adenocarcinoma’), and *mac*_*y*_ contains e.g. the weight concept. Everything is fine when *x*=*y*, which e.g. means that the weight concept belongs to the adenocarcinoma material. But when *x*≠*y* we found a false positive, which means that the weight concept references not to the adenocarcinoma. We introduce this problem herewith as *ECRI*.

During the XPath engineering, many false positives were found (caused by ECRI), but after many refinement cycles only one case was left in the result set of Q1, where the prostate flake weight was in the 13th item, while adenocarcinoma was not in the 13th item in the interpretation section; and one false positive was left in the result set of Q3, were ’Leiomyom’ was in the 11th item in the interpretation, but ’Uterus’ was in the 15th item of the specimen section.

**(2) PQCRI** Another reason for false positives occurred during the resolution of physical quantities to the bearing concept, which we will call PQCRI. For instance, one Search_Concept in Q3 is CM_Unit_in_Interpretation. During the manual review process it became clear, that this concept is not very precise because *cm* units occur in the interpretation section often without referencing a leiomyoma, but other tissue types or border distances. The solution, a gain of precision, can be enabled within the SOX approach by proximity searches, in detail by constructing a Composite_Term and connecting the Simple_TermLeiomyoma to the unit representing Simple_Termcm and adding the data property max_distance. A distance of ≈ 1-5 words seems to be meaningful, but the best concrete one has to be evaluated.

**Refinement circles** Variability of language yields an increasement of costs caused by cyclic refinements during the ontological engineering. In particular, much time was spend in refining Q1 and Q2 for increasing precision and recall. In one early query version, hundreds of false positives were found, because we searched only for the gram unit without a reference to flakes, which we introduced as PQCRI. As we increased the precision by the refinement of the query by a proximity search near the gram unit, we excluded many PEHRs. In brief, the refinement of the queries has shown, (1) the precise formulation of RQs is not easy, but ontologies can support; (2) in free text based records many writing variations are hindering a fast RQA.

### Coded language and standardization

Classification codes (like the Tumor Nodes Metastases (TNM) classification [29]) are used to face uncertainty of the NL, especially in the medical domain. When a classification code is available in the PEHR, queries should be based on classification codes.

We used openEHR-based, standardized XML, but we could have used also EN 14822 or even proprietary XML formats, regardless of the used NL. When the community comes to an agreement which EHR standard will be used in German Health Information Systems in future, not only the EHR would be interoperable, the usage of a standardized query language implies: queries can be interoperable too.

### Limitations and future work

The introduced ECRI and the latter PQCRI was unbound manually, which was time intensive. There are a lot of variations of enumeration styles, which are of course easy to understand for humans, but these variations are not instantly recognizable by machines. Another limitation is that Search_Terms are defined on a syntactic level, closely bound to the XPath syntax, e.g. we used XPath functions for matching word stems (→ “Querying PEHRs using XPaths” section). Since this only works with regular words in German, a deeper semantic understanding is necessary, also for preventing human errors during the manual review process.

Indeed, a human error was detected during the manual review process. For evaluation purposes, the Physical Quantity (PQ) had been transcribed a second time from the XML-snippets to a spreadsheet. In one case, there was a discrepancy of a value, which occurred during the transcription of the PQ on the spreadsheet. Consequently, the manual review process has to be automated for preventing human errors during the transcription of the values. This issue can be solved by pattern recognition, ontology extraction and SPARQL, which is a complex topic and could be described in another paper in the future.

**Archetype and XML_Structure relation** An automatic conversion of XML documents into a SOX XML_Structure tree is demandable; this would accelerate the query development in Protégé. X2OWL can generate an OWL ontology from an XML data source [30] and could be a good starting point.

**Domain experts, ontology editors and call for the clinician ontologist** Variety of language implies, that the definition of exact queries on PEHRs is a time consuming cyclic task; but at the same time, the ontology-based definition of such queries is promising time and cost savings. Since query engineering was done by an ontologist, the original plan, that domain experts can specify queries within ontology editors (→ Fig. 1) beside their daily clinical tasks, failed. But since the clinician has supported strongly the preparation process (*Understanding and Formalization of the Questions*), we could offer spreadsheets to the clinicians as input forms for the SO, because facilitated ontology engineering by the usage of spreadsheets [31–33] has much potential. However, our experiences during the refinement circles indicate, that ontological role allocations have to be proven in real clinical environments. In other words, when clinicians have not enough time beside their daily tasks for ontology engineering, it is perhaps time to think about a new clinical role, the *clinical ontologist*, who could manage all kinds of ontologies; the *clinical ontologist* could take care for the correct integration of terminologies like SNOMED, TNM or International Statistical Classification of Diseases and Related Health Problems (ICD), which will save costs, in particular during querying and answering processes.

## Conclusions

When PEHRs are section-structured by SBD and stored on an XML database, they can be exploited for RQA. The introduced Search Ontology XML extension connects Search Terms to certain parts in XML documents and enables an ontology-based definition of queries. We generated XPath expressions out of the ontology and proved practically, that *search ontology-based XPath engineering* can support RQA by the specification of complex XPath expressions without deep syntax knowledge about XPaths.

A precise automatic RQA on PEHRs requires coded language instead of NL. Since enumeration lists are used heavily for a linkage of material to other sections, retrieval of PEHRs by certain keywords in sections without a deeper semantic understanding of the content can be error prone. *Search ontology-based XPath engineering* can support, but not replace a manual review process. Since ontology engineering is time consuming, we suggest the contemplation about a new clinical role in hospitals, the *clinical ontologist*.

## Supplementary Legends

**Figure 1.** (1) The domain expert^1^ models the queries by the usage of SOX in Protégé. (2) Generation of XPath expressions out of the ontology. (3) Application of the generated XPath expressions. (4) Return of the relevant documents.

^1^In the original use case plan the domain expert was a clinician, but in practice is the Domain Expert an ontologist.

**Figure 2.** The snippet was cut to the necessary elements which are based on the openEHR-EHR-OBSERVATION.lab_test-histopathology.v1 archetype, which we want to address in the query in this paper. The doubling of the value tag is a result of the openEHR reference model, in practice the two value tags have different namespace declarations. In Q1 we are interested in PEHRs were adenocarcinoma occurs in the Overall_interpretation (black box in the listing) and a weight concept (underlined) in the near of prostate flakes (framebox).

**Figure 3.** Required XPath expressions for a search of EHRs which contains Adenokarzinom in the overall interpretation section.

**Figure 4.** SOX extends SO by additional classes and the *in* relation.

**Figure 5.** *Composite_Terms* are made up of *Simple_Terms*, related by the Object Property *has_part* and are constrained by the additional data property *max_distance*, which defines the worddistance between *Simple_Terms*, where *max_distance*=0 represents that one word immediately follows another word. Writing variations, synonyms of abbreviations of the *Simple_Terms* can be handled by the assignment of multiple labels to the concrete individual of a *Simple_Term*. The *Search_Concepts* are *described_bySearch_Terms*. *GFO top level concepts have been removed in the figure to increase readability.*

**Figure 6.** The Search Ontology XML Extension introduces the top level class *XML_Structure* and the relation *in* (dashed arrow). *GFO top level concepts have been removed in the figure to increase readability.*

**Figure 7.** The process starts with *I. Search ontology-based XPath Engeneering*, based on (M1) the RQ, and (M2) archetype-based PEHRs (yielding SOX.owl). After that, the *II. Automatic XPath Generation* process uses the query model (SOX.owl) and generates the required XPath expressions, which are added to the ontology as annotation properties. During *III. Fetching PEHR Snippets* relevant PEHR snippets are retrieved by applying the XPath expressions on an XML database. At the end, these XML snippets have to be reviewed during the *IV. Manual Review* process.

**Figure 8.** The XML_Structure tree is a HCG, which contains all elements in an XML file, which are relevant for queries.

**Figure 9.** The GFO top level concept Symbol_structure is refined by the XML_structure of the document (black background color) and Search_Term; the other GFO top level concept Concept is refined by Search_Concept and Search_Query. The Search_QueryQuest1_ProstateCancerGramCorrelation is subClassOf an anonymous class, which represented by a boolean expression containing Search_Concepts. E.g. is ICD-O-C-61_in_Localisation contained, which points to a class ICD-O-C-61 by the described_by relation. The instance of the class ICD-O-C-61 bears the classification string. In addition, the subClass description of ICD-O-C-61_in_Localisation contains the information about the XML part, where the instances of ICD-O-C-61 are expected, which is necessary for the XPath generation.

**Figure 10.** Class description of Quest1_ProstateCancerGramCorrelation, which is based on intersections and unions of classes, see Fig. 9 for an overview.

## Abbreviations

ECRI: Enumeration coreference resolution issue
EHR: Electronic health record
ETL: Extract transform load
GFO: General formal ontology
ICD: International statistical classification of diseases and related health problems
IR: Information retrieval
IRI: Internationalized resource identifier
NER: Named entity recognition
NL: Natural language
OWL: Web ontology language
HCG: Hierarchical concept graph
PEHR: Pathology electronic health record
PPIM: Pathology patient information model
OQL: Object query language
QA: Question answering
RQ: Research question
RQA: Research question answering
PQ; Physical quantity; PQCRI: Physical quantity coreference resolution issue
SNOMED: Systematized nomenclature of medicine
SBD: Section boundary detection
SO: Search ontology
SOX: Search ontology XML extension
SPARQL: SPARQL protocol and RDF query language
SQL: Structured query language
TNM: Tumor nodes metastases
XML: Extensible markup language
XSLT: Extensible stylesheet language transformation
